# 

**Published:** 1965-07

**Authors:** 


					A
r\ Or^ ^ 2-^ July 1965
NATIONAL LIBRARY OF MEDICINE
fiECD. r: > 1CPt-
JUL4 o iy30
iTc
VOL-!SSUE_
/4L
INCORPORATING THE MEDICAL JOURNAL OF THE SOUTH WEST
Vol 80 (iii) No 297

				

